# Supplementary figures and images for: Modeling the ecologic niche of plague in sylvan and domestic animal hosts to delineate sources of human exposure in the western United States
Source: PeerJ. 2015 Dec 14;3:e1493. doi: 10.7717/peerj.1493 (PMC4690378; doi:10.7717/peerj.1493)

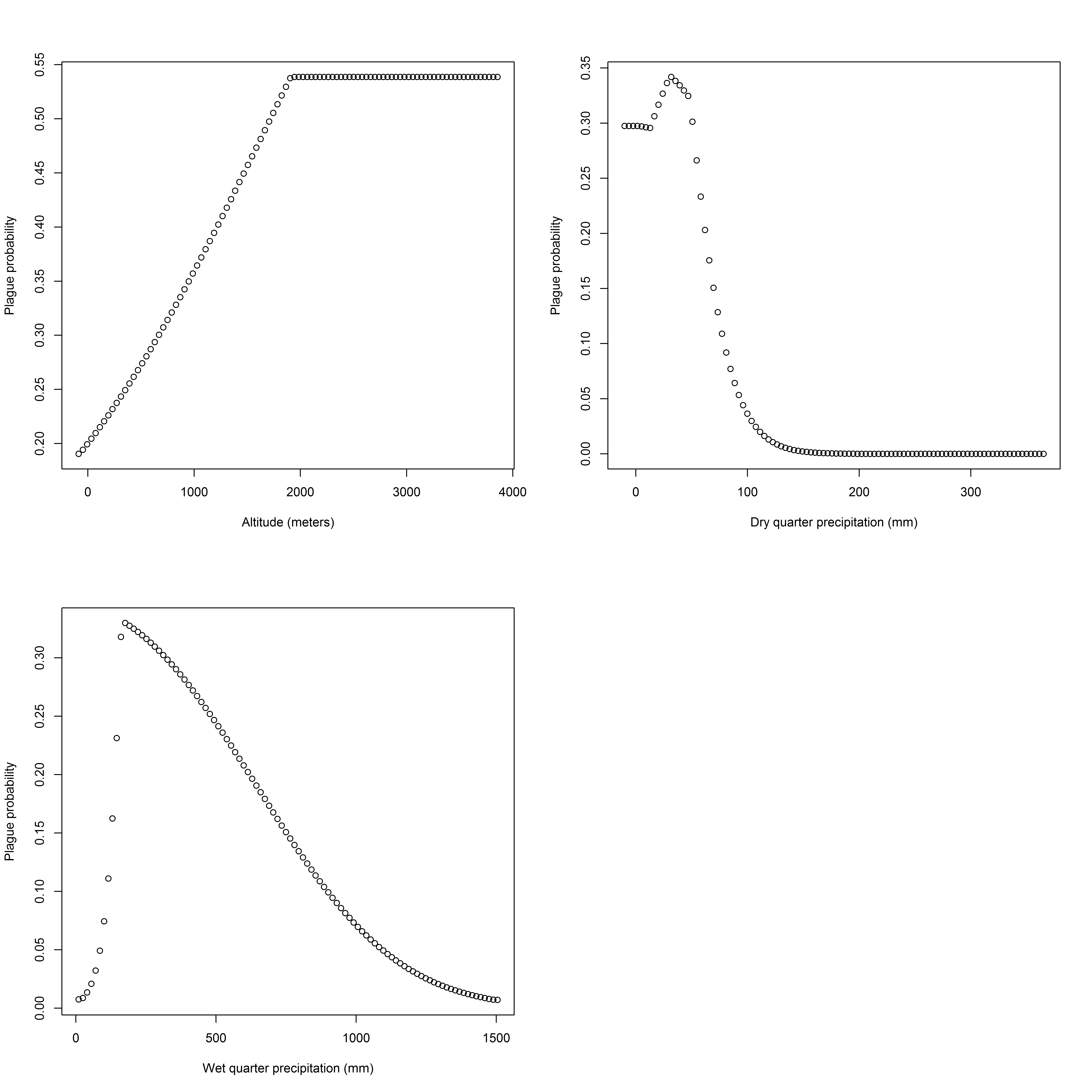

Supplement: Figure S1 [file peerj-03-1493-s001.png]

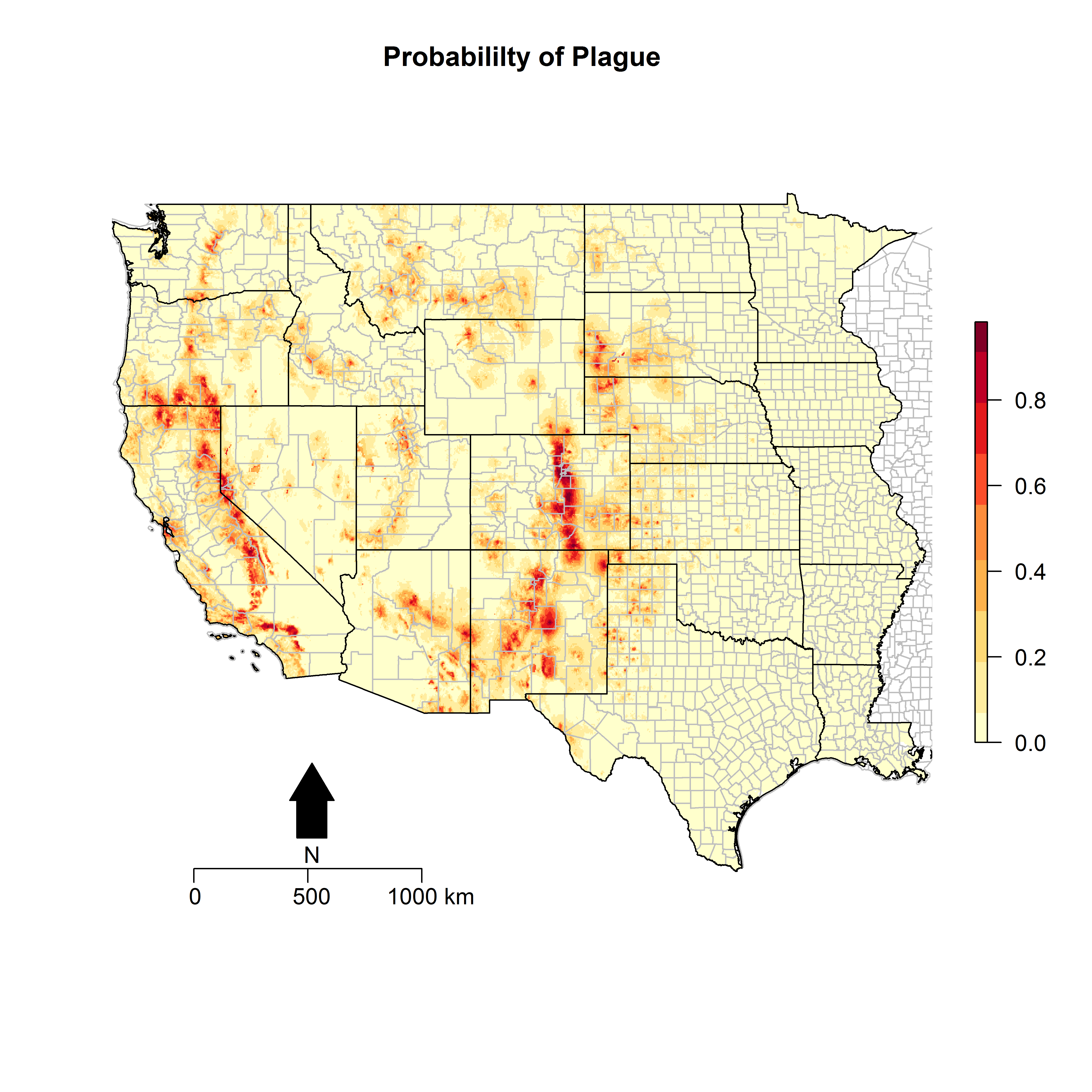

Supplement: Figure S2 — These risk surfaces are based on the ecologic niche of animal plague as derived from the Maxent model. All predictions are based on landscape variables aggregated to 5 km2 resolution. [file peerj-03-1493-s002.png]
